# Free Flap Selection and Outcomes of Soft Tissue Reconstruction Following Resection of Intra-oral Malignancy

**DOI:** 10.3389/fsurg.2019.00053

**Published:** 2019-12-20

**Authors:** Adam M. H. Young, Sarah Bache, Nicolas Segaren, Suzane Murphy, Jane Maraka, Amer J. Durrani

**Affiliations:** Department of Plastic Surgery, Addenbrooke's Hospital, University of Cambridge, Cambridge, United Kingdom

**Keywords:** intra-oral, free-flap, outcomes, malignancy, RFFF, ALTFF

## Abstract

**Introduction:** Surgery to resect intra-oral malignancy is a well-established mode of primary treatment. The tissue requirement in this area is for a thin, pliable flap with minimal bulk and this has historically been provided by free tissue transfer with a radial forearm free flap (RFFF). More recently, a role for the anterolateral thigh free flap (ALTFF) has been described, although in populations with a westernized diet, body habitus may preclude use of an ALTFF due to flap thickness, relative to a radial forearm free flap.

**Methods:** An analysis of data was performed retrospectively for 90 consecutive patients with intra-oral malignancy, requiring immediate soft tissue reconstruction by the senior author, at Addenbrooke's Hospital between July 2008 and April 2016. Cases requiring bony reconstruction were excluded. Data on patient age, sex, indication for surgery, tumor location and defect type, complications, success rates, and length of stay were recorded.

**Results:** The majority of patients received an ALTFF (*n* = 56) with 38% receiving a RFFF (*n* = 34). Surgical resection took place in the floor of the mouth most frequently. These were closed with ALTFF and RFFF in 41 and 28 occasions, respectively. A success rate of 97% was observed in the RFFF group; 1 flap developed partial necrosis and required complete revision. In the ALTFF group, there was a 100% flap success rate. ALTFF usage resulted in a reduction in the number of intraoperative (*p* = 0.021) in addition a reduction in the number of days in ITU (*p* = 0.01) and post-operative clinic visits (*p* = 0.025).

**Conclusion:** We present a series that used predominately the ALTFF to reconstruct intra-oral defects following resection of squamous cell carcinoma in a Western population. The results demonstrate that this treatment can produce at least as comparable results as to the use of a RFFF repair in this population, whilst avoiding the donor site morbidity and aesthetic compromise of a RFFF.

## Introduction

The frontline treatment for intra-oral carcinomas are primarily surgery-based therapies (1). In instances of ablative surgery or in the case of small/medium sized defects there are a number of surgical options including primary closure or local flaps. However, as the size of the defect increases so does the challenge of reconstruction. When a lesion also involves the tongue, the optimal reconstructive method should combine satisfactory structural cosmesis with good restoration of function of speech and swallowing. Opinions around optimal flap choice continues to be divided a study by Husso et al. (2) demonstrated that the number of different flap types and flap combinations increased between 1995 and 2012 from 15 to 24 in the 5 year periods analyzed.

Radial forearm free flap (RFFF) has become the favored option for correcting defects after ablative tumor surgery of the oral cavity. Introduced as the “Chinese flap” by Yang et al. (3) in 1981 and Song et al. (4) in 1982, it has multiple advantages (3, 4). Based on its long high-caliber pedicle, reliability of anatomical structures, and overall the ease of grafting qualifies it as an ideal flap for microsurgical reconstructions. Nevertheless, the burden of donor-site morbidity and cosmesis among RFFF patients has left surgeons searching for more appealing reconstructive options.

The anterolateral thigh (ALT) free flap was first described by Song et al. (5) and Wei et al. (6) proposed that the ALT failure rate was <2%, and subsequently concluded that the ALT flap was superior to most other flaps for soft tissue. Based on the availability of a long pedicle with a suitable vessel diameter, low donor site morbidity and versatility in design the ALT flap is favored for reconstructive microsurgery. Its adoption ranges from reconstruction of the head and neck, upper and lower extremities, and the trunk and breast (7–11), and momentum is gathering on the use in tongue reconstruction (12–15).

ALTFF has been most widely adopted in the eastern countries of Japan and Taiwan with many being skeptical of its use in European populations due to the fat distribution and muscle bulk in this cohort (16, 17). Here in, we report our experience with the ALT flap for defects of the tongue and floor of the mouth in a western population, highlighting the reasons for its versatility and benefits.

## Methods

### Data Collection and Analysis

Data were prospectively collected from July 2008 until April 2016 and documented in the Addenbrooke's Hospital, Plastic Surgery, free tissue transfer log book. Data was collected in accordance with Addenbrooke's Hospital clinical audit guidelines and reviewed retrospectively. To evaluate the advantages of ALT flap more clearly, we made a comparison between the group receiving ALT and a group in which all patients received radial forearm flap (RFF) reconstruction for defects in the tongue, floor of mouth and retromolar trigone.

The operation-based characteristics that were recorded and compared between ALTFF and RFFF included clinical details on age, sex, indication for surgery, type and location of malignancy, flap success rate, and number of operative revisions, flap size, primary or secondary reconstruction, operation time, microvascular complications, return to theater rate, duration of stay in intensive care unit (ICU), prolonged stay on ICU (longer than 25 h), length of hospital stay (LOHS) more than 14 days, and outpatient intervention within 30 days of hospital discharge. Operative revisions, prolonged stay on ICU, and LOHS over 20 days were identified as complications; such complications were documented and compared between both flap types.

Details recorded and entered into a Microsoft Office Excel 2010 (Microsoft, USA) spreadsheet.

### Surgical Intervention

In all cases, a dual-team approach was implemented to reduce total procedure time. Surgical resection and neck dissection was carried out by the referring speciality for each case (ENT, Maxillofacial Surgery), under general anesthesia. Harvesting and microvascular repair of the ALT/RFF was simultaneously performed or supervised by the senior author (AJ Durrani) in the Department of Plastic Surgery, Addenbrooke's Hospital, University of Cambridge, Cambridge, UK.

For each patient, the choice of flap type was based on surgeon experience, defect size, and the patient's characteristics including body habitus, donor site suitability and comorbidity. Perforator detection, planning and dissection of the ALT flaps were performed as described historically (7, 18, 19). Only one ALT flap required primary thinning. RFFF were raised as described by Yang et al. (1) and Song et al. (3) and harvested in the subfascial plane.

After flap harvest was completed, each flap was allowed to perfuse *in situ* until completion of the ablative surgery and recipient vessel preparation. This was followed by pedicle division, flap inset and microvascular anastomoses were immediately performed to minimize ischemia time. As described previously, this procedure allows the extended monitoring of the vessel anastomoses prior to wound closure (20). In the case of early thrombosis, spasm, kinking, bleeding from the vessel, or other signs of flap ischemia, the surgeon was able to perform an intra-operative revision.

### Statistical Analysis

SPSS 13.0 software (SPSS Inc., Chicago, IL) was used to perform statistical analysis and create diagrams. Continuous variables were analyzed by the non-parametric Mann-Whitney *U*-test for the comparison of two independent samples. Binary variables were analyzed using Fisher exact test (sex, defect type, primary/secondary reconstruction, stay on ICU for more than 25 h, stay in hospital for more than 20 days, flap thinning, chosen donor site). Categorical parameters were reduced to binary variables as a consequence of the limited number of cases (ASA class: 1/2, 3/4; revisions: none, 1, or more). All *P*-values given are unadjusted, 2-sided, and subject to a local significance level of 5%.

## Results

### Patient Demographics

Ninety patients underwent soft tissue reconstruction following resection of intra-oral malignancy in Addenbrookeio Hospital between July 2008 and April 2016. Of these the majority of patients received an ATTFF (*n* = 56) with 38% receiving a RFFF (*n* = 34). The median age was 63 years in the RFFF population compared to ALTFF with a median age of 62 years. The RFFFs were performed more frequently on female patients (61%) compared to ALTFFs which were only performed on females on 25% of procedures.

BMI was 26.7 kg/m^2^ in RFFF and 24.3 kg/m^2^ in the ALTFF group (*p* = 0.02). The full demographical characteristics are displayed in Tables 1, 2.

**Table 1:** Patient and operation based characteristics Tested with Fisher exact test, percentages related to all patients of 1 group.

	**RFFF** ** (*n* = 34)**	**ALTFF** ** (*n* = 56)**	***p*-value**
	**Count (%)**	**Count (%)**	
**Sex**			
Male	14 (41)	42 (75)	
Female	20 (59)	14 (25)	
**Histology**			
Squamous cell carcinoma	34 (100)	52 (93)	
Merkel cell	0	1 (2)	
Metastasis	0	3 (5)	
**Primary tumor location**			
Lower oral cavity	30 (90)	51 (92)	
Cheek	3 (10)	1 (2)	
Upper Jaw	0	4 (6)	
**Defect type**			
Intraoral	34 (100)	53 (94)	
Combined	0	4 (6)	
**ASA group**			
I/II	68	71	0.652
III/IV	32	29	
Overall success	97	100	0.156
**Complications**			
Overall failure	3	0	
Intraoperative time (mins)	153 ± 16	168 ± 52	
Intraoperative revisions	13	8	0.021
Postoperative revisions	12	10	0.784
Wound healing donor site	15	2	0.001
Preoperative radiation	10	2	0.001
ICU days > 24 h	16	6	0.01
Length of stay > 14 days	16	17	0.452

**Table 2:** Patient- and operation-based characteristics.

	**RFFF**		**ALTFF**		***p*-value**
	**Median**	**Range**	**Median**	**Range**	
Age (years)	63	30–87	62	18–88	0.652
Flap volume (mm^3^)	31.2 × 10^3^	25.2–169 × 10^3^	39.5 × 10^3^	14.7–247.5 × 10^3^	0.018
Flap thickness (mm)	8.2	6.8–12.2	14.6	12.4–16.2	0.001
Ischemic time (min)	64	31–101	76	21–126	0.063
Stay in ICU (hours)	23	13–50	16	8–32	0.003
Follow up visits^*^	2	0–3	1	1–2	0.025

* *Follow up visits measured until 6 months*.

### Intra-oral Reconstructed Sites

The majority of cases were to reconstruct defects after resection of squamous cell carcinoma (SCC), with only one case of Merkel cell carcinoma and two metastatic cases being covered by an ALTFF. Surgical resection took place in the floor of the mouth most frequently. These were closed with ALTFF and RFFF in 41 and 28 occasions, respectively. Hemiglossectomy defects were repaired with 19 ALTFF and 16 RFFF and total glossectomy in 7 and 8 instances using ALTFF and RFFF, respectively. In maxilla reconstructions ALTFF was preferred in 6% of occasions, RFFF was not used.

Buccal defects were replaced RFFF in 10 cases compared to 2 cases in ALTFF. Combine intra and extra oral defects were repaired using ALTFF only (2 cases).

### Outcome and Peri-Operative Characteristics

The procedure was successful in 97% of RFFF procedures; 1 flap developed a partial necrosis and required revision. In the ALTFF group, there was complete success (*p* = 0.156). Within the RFFF patients, 13% were returned to theater and underwent operative revision, compared with 8% among ALTFF patients (*p* = 0.021). Five patients who received a RFFF developed a donor site infection, 2 with exposed tendon. In the ALTFF group 1 episode of delayed wound healing was identified (*p* = 0.001). The median flap volume was 39.5 × 10 mm^3^ in ALTFF and 31.2 × 10 mm^3^ in RFFF (*p* = 0.018). The median ischemic time was prolonged in ALTFF reconstructions (76 min) when compared with RFFF (64 min) reconstructions (*p* = 0.063).

There was a significant difference between the groups with regards to the length of ICU admission where the median stay for RFFF was 23 h compared to 16 h in ALTFF (*p* = 0.003) in patients admitted to the unit. Only 6% of ALTFF patients were actually admitted compared to 16% of RFFF patients (*p* = 0.01). Prolonged hospital admission was not statistically significant between patient groups (*p* = 0.452). Of the 31 RFFF patients, 5 stayed over 14 days in hospital (16%), compared with 8 ALTFF patients (17%). Within 30 days of hospital discharge, the RFFF patients had a significantly higher rate of outpatient clinic reviews/interventions (*p* = 0.001). Patient and operation variables are detailed in Table 2.

### Complications

In the RFFF group, four surgical procedures followed the first operation, whereas five surgical procedures occurred in the ALTFF group. Of the further procedures in the RFFF group, 3 (75%) were flap-related, whereas there were no flap related complications in ALTFF patients. One RFFF returned to theater for evacuation of a neck hematoma whereas in the ALTFF group 1 patient required secondary wound closure, 1 required a hematoma evacuation, 1 required a thoracic duct repair, and 2 patients required tracheostomy (Table 3).

**Table 3:** Post-operative revisions.

	**RFFF**	**ALTFF**
	**Count**	**Count**
**Number of revisions**		
1	6	3
2	0	0
3	0	0
Total	6	3
**Operative revision, head and neck related**		
Secondary wound closure	0	1
Tracheostomy	0	2
Hematoma evacuation	1	2
**Operative revision, flap related**		
Flap failure, new flap	1	0
Resetting of flap	1	0
Venous revision	1	0
Total	3	0

Overall, 16% of the RFFF patients and 17% of the ALTFF patients had a prolonged hospital stay. Among these, 27% of RFFF patients experience a surgical complication and no medical complications and in ALTFF patients 10% experienced a surgical complication with 8% of patients being managed for medical complications. Of the surgical complications, the primary reasons were associated with the original neck dissection in 89% of RFFF patients and in all of ALTFF patients. Fifteen percent of RFFF patients experienced a donor site infection with only 2% of infections in ALTFF patients (Table 4).

**Table 4:** Reasons for prolonged stay on intensive care unit >24 h.

	**RFFF**	**ALTFF**
	**Count**	**Count**
**Surgical complications**	9	5
**Head and neck related**	8	5
Swelling	1	1
Hematoma	2	2
Sepsis, fever	0	1
Surgical site infections	5	1
**Flap related**	1	0
Flap Instability	1	0
**Medical complications**	0	4
Pneumonia	0	2
Pulmonary oedema	0	1
Atrial fibrillation	0	1

## Discussion

There is evolving debate over whether RFFF remains the flap of choice to repair a defect following intra-oral malignancy resection. The ALTFF is widely used throughout eastern practice however its popularity in Europeans is limited (16, 17). The primary reason cited for the lack of uptake is the difference in baseline body habitus (17), with western populations tending to have a higher fat and muscle bulk making the ALTFF disproportionately thick to apply to the defect. The ALTFF was the preferred option for repair. RFFF was primarily selected when the thigh appeared thicker and unsuitable for repair. Despite the fact that BMI of patients being similar between both groups the habitus of patients can vary widely, particular in cancer patients. While it is accepted that only one ALTFF was thinned (21, 22) and that more could have been made suitable with this process (23), the RFFF was the preferred option in the case where the ALTFF was felt too thick from the outset. For the first time to our knowledge, we present a European population of patients requiring treatment of a deficit after intra-oral malignancy resection who have been predominately treated using ALTFF.

The advantages of the ALTFF over the RFFF, such as the preservation of major vascular structures and primary donor-site closure demonstrates why is it of value when selecting tissue flaps (20, 24, 25).

The study was designed to review the objective peri-operative criteria of RFFF and ALTFF selection in oral cavity reconstruction and observe the post-operative complications associated with the procedures. With regards to demographics the ALTFF group were predominately male and the RFFF predominately female which is in keeping with earlier observations by Yu et al. (26). RFFF were used only to cover defects from squamous cell carcinoma resection whereas ALTFF were used to cover defects left after Merkle cell carcinoma and metastatic disease. ALTFF were also used to cover more extensive defects in the cheek and lower jaw which were not possible to cover with a RFFF (6, 26).

Although only one RFFF failed all ALTFF healed successfully. A similar pattern was observed with intraoperative revision rates. Thirteen intraoperative revisions were required in the RFFF cohort compared to only 8 in the ALTFF cohort. After which four (12%) RFFF required post-operative revisions compared to 5 (9%) ALTFF procedures. These findings compare to that of Loreti et al. (27) who described success rates of 100 and 94.2%, respectively, for ALTFF and RFFF however the cohort was significantly smaller than the group we describe and predominately RFFF. This was also described by Huang et al. (28) however only 21 ALTFF and 20 RFFF procedures had been performed. Our results demonstrate increased success compared to that Nakatsuka et al. (29) where complete survival was reported in 90.8% of thigh flaps and 95.8% of forearm flaps.

Interestingly in our cohort we found that the both donor site complications and ICU admission days were minimized by using the ALTFF. It is unclear why this is the case, perhaps it is related to the surgical confidence in the ALTFF and possible that the RFF was observed for longer in ICU. Where the ICU stay in these patient groups appear long, it is representative of the physiological reserve of the patients who are treated. Head and neck cancers are predominately associated with heavy use of tobacco and alcohol and significant comorbid disease. As a result the complex nature of the microvascular procedures involved can test the physiological reserves of such patients and result in extended stay on ICU and an overall duration of hospital admission (16).

Comparison of the operation time between cohorts demonstrated a longer operation time for ALTFF, however this did not reach significance. This might be a result of early implementation of ALTFF when larger resections are anticipated with anterolateral thigh flaps being the procedure of choice for larger resections. We described a hospital stay longer than that by Clark et al. (30) who describe an average stay of 15 days. However, this may be accounted for by the fact 80% of that particular cohort were treated with RFFF.

The number of surgical revisions in the cohort adds testament to the complexity of the operations, particularly, in the more extensive cases that required ALTFF to repair the deficit. A complication rate of 24% is significantly improved compared that to the largest series reported (30). More in keeping with that of Rosenberg et al. (31) who reported 29% of complications. As observed with previous series the most frequent surgical complication was hemorrhage in the primary surgery (32). This was less frequent in the ALTFF cohort. Within the ALTFF cohort the most common surgical complication was a requirement for a surgical tracheostomy because of the requirement for prolonged respiratory weaning from the ventilator. It is unclear why ALTFF use had a more favorable outcome in regards to complication rates. Given that more flap revisions and infections were observed in the RFF cohort, it could be suggested that the perfusion of the free flap was better after surgery in the ALTFF group. Additionally, it is important to note that significantly more RFF patient underwent preoperative radiotherapy. Those that did, accounted for one intra-operative revision, one post-operative resetting of the flap and three surgical site infections.

The adverse effects of radiotherapy are observed from the acute phase of treatment (33, 34) and are directly linked to the radiosensitivity of the anatomy being targeted, the volume of normal tissue irradiated, the total dose and the rate of dose accumulation (21). Side effects are most evident in rapidly proliferating tissues, such as the skin, mucosa and bone marrow, but can arise in any organ. There effects can extend from the initial few days to beyond 3 months after treatment and can be progressive. Side effects are usually a result of mass cell death within irradiated organs and secondary ischemia as a result of the effects of radiation on small vessels and because of perturbed inflammatory and repair responses (22).

It is worth noting that the selection of both RFFF and ALTFF were evenly distributed chronologically across the series. As such, the improved survival, shorter length of stay and diminished revision rate is likely to be genuine rather than a result of temporal artifact. This data demonstrates that with correctly selected patients the ALTFF can provide excellent cover for large defects in western populations. Nevertheless, the average male body habitus is more likely to have thin, pliable tissue at the thigh donor site compared to women. In addition to this, the number of intra-operative revisions is a testament to good clinical practice and patience to observe that appropriate correction of the free tissue transfer can significantly reduce the post-operative revision rate. In addition to this work by Zhang et al. (33) demonstrated that the use of ALTFF for intra-oral reconstruction can improve quality of life.

## Conclusion

In conclusion, this study presents the treatment of intraoral defects in a European population using predominately ALTFF reconstructions. The RFFF is a very reliable flap for microsurgical reconstructions of the oral cavity. In our study, the ALTFF was used with a lower number of complications and reduced both in hospital stay (ITU and ward) and also reduced the number of post-operative clinic appointments. This study encourages the adoption of the ALTFF despite its variable anatomy and the requirement for greater surgical experience.

## Author Contributions

AY, NS, and SB did the data analysis and wrote the paper. SM and JM performed the primary data collection. AD designed the study and supervised the project.

### Conflict of Interest

The authors declare that the research was conducted in the absence of any commercial or financial relationships that could be construed as a potential conflict of interest.

## Abbreviations

ALT: anterolateral thigh
RFFF: radial forearm free flap
SCC: squamous cell carcinoma.
